# Epigenetic blind spots – the role of DNA methylation dynamics in stem cell‐based models of embryogenesis

**DOI:** 10.1002/1873-3468.70350

**Published:** 2026-05-19

**Authors:** Sara Canil, Anna Corrà, Annalisa Scapolatiello, Chengxiang Qiu, Gianluca Amadei

**Affiliations:** ^1^ Department of Biology University of Padova Italy; ^2^ Department of Molecular and Systems Biology Dartmouth College Hanover NH USA

**Keywords:** DNA methylation, embryogenesis, embryos, epigenetics, stembryo

## Abstract

Embryo‐like structures, or stembryos, complement embryo research in innovative ways and provide an alternative when embryo utilization is ethically or technically limited. These models, however, are hindered by experimental variability and limited developmental potential, whose causes are still not fully understood. Establishment of appropriate DNA methylation patterns is crucial for mammalian development but characterization of DNA methylation in stembryo models has been largely overlooked. This Perspective manuscript highlights this knowledge gap and suggests that characterization of DNA methylation, and in the future also of other epigenetic modifications, should become standard practice in stembryo model characterization. Available datasets indicate that differences in DNA methyltransferase expression between stembryos and embryos exist, thus suggesting an avenue of investigation to further improve these models.

## Graphical abstract

Embryo‐like structures (stembryos) are an innovative tool, but they are hindered by experimental variability and limited developmental potential. DNA methylation is crucial for mammalian development, but its status in stembryo models is poorly characterized. This Perspective suggests that characterization of DNA methylation should become standard practice in stembryo model characterization because differences in DNA methyltransferase expression between stembryos and embryos exist. Created in biorender. Ghirardo, R. (2026) https://BioRender.com/fuwcgl9.

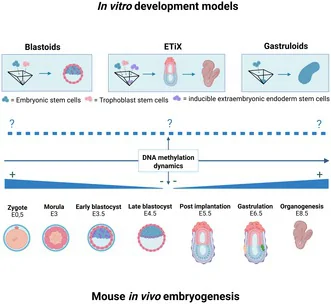

## Abbreviations

**2i**, two inhibitors (MEK inhibitor + GSK3 inhibitor)

**5fC**, 5‐formylcytosine

**5caC**, 5‐carboxylcytosine

**5hmC**, 5‐hydroxymethylcytosine

**5mC**, 5‐methylcytosine

**ADD**, ATRX‐DNMT3‐DNMT3L

**ART**, assisted reproductive technologies

**ATAC‐seq**, assay for transposase‐accessible chromatin using sequencing

**B6**, 129SV/Bl6 line

**CEPT**, chroman 1, emricasan, polyamines, and trans‐ISRIB

**CMML**, myelomonocytic leukemia

**CpGi**, CpG islands

**CS6**, Carnegie stage 6

**DKO**, double knockout

**DVE/AVE**, distal/anterior visceral endoderm

**E(number)**, embryonic day (number)

**EPI**, epiblast

**ESCs**, embryonic stem cells

**ETiX**, ETiX‐embryoids

**ExE**, extraembryonic ectoderm

**FGF**, fibroblast growth factor

**IB10**, E14‐IB10 line

**ICM**, inner cell mass

**ICR**, imprinting control regions

**K4**, lysin in position 4

**KO**, knockout

**LIF**, leukemia inhibitory factor

**MDS**, myelodysplastic syndrome

**PE**, parietal endoderm

**PrE**, primitive endoderm

**PSCs**, pluripotent stem cells

**PWWP**, proline‐tryptophan‐tryptophan‐proline

**RFTS**, replication foci targeting sequence

**Sc‐Fv**, single‐chained variable fragments

**scRNAseq**, single cell RNA sequencing

**sEmbryo**, advanced postgastrulation embryo‐like models

**TDG**, thymine DNA glycosylase

**TE**, trophectoderm

**TET**, ten‐eleven translocation

**TF‐SEM**, transgene‐free embryo models

**TGFβ**, transforming growth factor β

**TR**, triple germ layer reporter

**TSCs**, trophoblast stem cells

**UBL**, ubiquitin‐like

**VE**, visceral endoderm

**XEN**, extraembryonic endoderm stem cells

## Overview

Embryo‐like structures, or stembryos, are stem cell‐based models of embryonic development that bridge the gap between animal models and traditional two‐dimensional stem cell cultures. Stembryos hold the promise to reduce our reliance on traditional animal models (i.e., mice and rats) and to enable modeling of embryonic development and perturbations in contexts in which embryos are not available, impractical to obtain (e.g., primates) or subject to ethical and technical limitations (e.g., humans).

These models, however, are far from perfect. In fact, only a subset of stembryos generated in any given experiment correctly acquires a physiological embryonic morphology with the potential to progress through the entire culture protocol. However, the reasons underlying this limited efficiency and pronounced structure‐to‐structure variability are still not fully understood.

It is important to note that multiple factors are likely to contribute to this variability, including genetic background, stem cell state, culture conditions, signaling environments, and epigenetic regulation. Epigenetic regulation represents one potential, and largely unexplored, contributor to these differences. Among different types of epigenetic regulation, DNA methylation is known to play a key role in mammalian development, and it is the focus of this Perspective article because of its potential relevance to stembryo models.

Work on stembryo models to date has largely focused on the characterization of morphology, lineage marker expression, and transcriptomic analysis. By contrast, their DNA methylation landscape—both at the level of the starting stem cell populations and during stembryo development—has received comparatively little attention.

This Perspective article aimed to highlight this underexplored gap in the field and to argue that epigenetic regulation, and in particular DNA methylation dynamics, should be more systematically considered when evaluating the developmental fidelity of stem cell‐based embryo models. By integrating evidence from published datasets and recent studies, we discuss how differences in the expression and regulation of DNA methyltransferases may affect DNA methylation and in turn contribute to experimental variability and limited developmental potential in current stembryo systems. Rather than proposing DNA methylation as a singular explanatory mechanism, we suggest that its characterization should become a standard component of stembryo model assessment, alongside morphological, transcriptional, and functional analyses. Looking ahead, targeted interventions may offer one possible route to improving developmental fidelity and reproducibility in these systems.

## The mysterious mammalian development

The development of mammalian embryos relies on the interactions between their constitutive tissues: the epiblast, the extraembryonic ectoderm and the extraembryonic endoderm, precursors respectively to the fetus, the embryonic portion of the placenta and the yolk sac [1, 2]. After fertilization, the newly formed zygote undergoes a series of growthless divisions, called cleavages, whose function is to establish the founder cells of these three lineages and culminates in the formation of the blastocyst, which then proceeds with implantation into the uterus [3].

Because the stages following implantation are hidden from view inside the body of the mother, these have been referred to as the ‘black box’ of development [4]. Research with mouse and rat embryos, and recently with primate and human embryos thanks to the establishment of *ex utero* culture protocols [5, 6, 7], is finally unraveling the complex series of steps controlling blastocyst morphogenesis as it acquires its postimplantation conformation: the egg cylinder of the mouse and the bilaminar disk of primate and human embryos. Our current knowledge of early mammalian development could not have been obtained without model organisms, but these are costly, low‐throughput, and, with respect to human embryos, riddled with ethical and technical limitations [8, 9].

## Reconstitution of mammalian postimplantation development *in vitro* using stem cells

Establishment of stem cells from embryos has enabled over the last few decades to model discreet aspects of early development in a dish. Embryonic stem cells are derived from the epiblast and they are pluripotent, hence they can give rise to all the cells in an organism. Naive pluripotent stem cells resemble the pre‐implantation epiblast and have the potential to generate ectoderm, mesoderm, endoderm, and the precursors of the germline [10]. In order to retain these properties, mouse‐naive pluripotent stem cells are grown in defined culture conditions known as 2i/LIF, a chemically defined, serum‐free culture medium comprising leukemia inhibitory factor (LIF), which is an activator of the STAT3 pathway, and two selective small molecule inhibitors (2i): PD0325901, a MEK inhibitor, and CHIR99021, an inhibitor of glycogen synthase kinase 3 [11, 12, 13]. Mouse naive embryonic stem cells can also be propagated on a layer of feeder cells in culture medium supplemented with fetal bovine serum, which introduces undefined and variable components, and LIF [14]. These latter conditions, however, promote heterogeneity and spontaneous differentiation [15]. Primed pluripotent stem cells instead resemble more closely the postimplantation epiblast and upon differentiation they upregulate markers associated with germ layer lineage commitment [16, 17]; successively, they can differentiate into all embryonic germ layers, but are unable to give rise to primordial germ cells [18, 19, 20]. Naive and primed pluripotent stem cells share several core pluripotency transcription factors, but they express specific ones for the maintenance of one state or the other [10]. Upon removal of 2i/LIF, naive pluripotent stem cells quickly downregulate the naive pluripotency network, and after a short time window in which addition of 2i/LIF is sufficient to revert them, they irreversibly acquire primed pluripotency and become competent for germ layer differentiation [17]. Unlike mouse naive embryonic stem cells (ESCs), mouse primed ESCs are derived and propagated *in vitro* by activation of the FGF and TGFβ signaling pathways by addition of FGF2 and activin A [17].

From the extraembryonic tissues of the blastocyst, which are the trophectoderm and the primitive endoderm, it is possible to derive trophoblast stem cells (TSCs) and extraembryonic endoderm stem cells (XEN), respectively [21, 22]. Similar to embryonic stem cells, both serum‐based and serum‐free media exist for the maintenance and propagation of TSCs and XEN cells [22, 23, 24]. It is also possible to transdifferentiate embryonic stem cells into TSC‐like or XEN‐like stem cells by transient expression of specific transcription factors or chemically [25, 26, 27].

Stem cells have been widely utilized in 2‐dimension and 3‐dimension *in vitro* assays to study specific aspects of development and recently, a new milestone has been reached with the establishment of stem cell‐based embryo models, or stembryos [28, 29, 30, 31, 32, 33, 34, 35, 36, 37]. At present, this is a flourishing and expanding field of research, and new models, or variations of the existing ones, are constantly reported. This Perspective article does not aim to provide an exhaustive review of all available stem cell‐based embryo models; for comprehensive and in‐depth analyses of this rapidly expanding field, we refer the reader to several recent reviews [9, 38, 39].

**Fig. 1:**
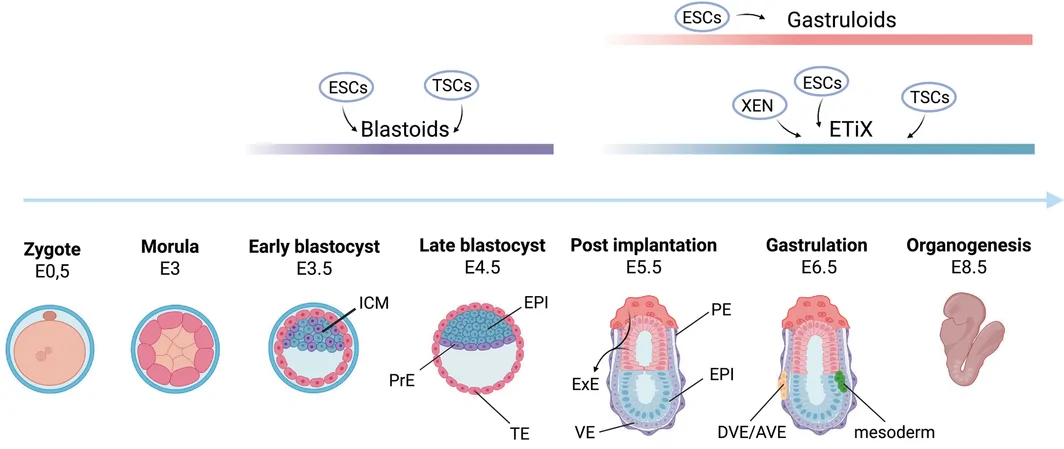
Schematic representation of mouse embryonic development. On the top, each line identifies the models in discussion with the starting cell types and the embryonic developmental stage they represent. DVE/AVE, distal/anterior visceral endoderm; EPI, epiblast; ExE, extra‐embryonic ectoderm; ICM, inner cell mass; PE, parietal endoderm; PrE, primitive endoderm; TE, trophectoderm; VE, visceral endoderm. Created in biorender. Ghirardo, R. (2026) https://BioRender.com/fuwcgl9.

Here, we focus on a subset of foundational stembryo models that illustrates key principles and current limitations. These models, which are gastruloids, blastoids, and ETiX‐embryoids (ETiX), represent distinct yet widely adopted approaches to modeling early mammalian development, differing in starting cell types, cellular complexity, and the developmental stages they aim to capture [40] (Table 1 and Fig. 1). While their specific implementations and efficiencies vary across laboratories and protocols, many of the conceptual challenges that we will highlight in this Perspective article, such as variability and incomplete developmental progression, are largely shared across related models. We therefore use these models as illustrative case studies rather than as an exhaustive representation of all stembryo models.

**Table 1:** An overview of key stembryo models.

Model	Starting cell types	Stage representing	Characteristics	Limitations	References
Gastruloids	ESCs	From gastrulation to early organogenesis (E6.5–8.5)	Develop mesoderm and endoderm lineages; posterior/trunk genetic program establishment	Lack of extraembryonic tissues and anterior structures	[33, 37, 41, 42, 43, 44]
Blastoids	ESCs, TSCs	Blastula (E3.5–4.5)	Display accurate blastocyst morphology and gene expression compared to *in vivo* embryos	Only represent early stages of embryonic development; Inefficient acquisition of post‐implantation morphology; fail to develop after transfer in the uterus	[9, 34, 45, 46, 47, 48]
ETiX	ESCs, TSCs, XEN	From post implantation to early organogenesis (E5.5–8.5)	Display accurate morphology, gene expression and cell type identities compared to *in vivo* embryos	Low efficiency; Roller culture required for post‐gastrulation development and organogenesis	[28, 49, 50, 51]

### Gastruloids

Gastruloids are perhaps the simplest model, since they require only embryonic stem cells as their starting point [37]. In this model, a few hundred embryonic stem cells are aggregated and then pulsed with a WNT signaling agonist to trigger a process of axial elongation. Over the course of 5 days as they elongate, they develop cells of mesoderm and endoderm, and establish a genetic program of the posterior/trunk region of the mammalian embryo; therefore lacking anterior structures such as the head. Since the publication of the original protocol, many modifications have been reported [41]. For instance, addition of exogenous factors during culture can be exploited to bias gastruloids toward a specific fate [33], and their latent morphogenetic potential can be revealed by embedding them in an exogenous extracellular matrix, such as Matrigel, in which they form somites flanking a neural tube [31]. Gastruloids can also be grown two‐dimensionally as micropatterns by plating ESCs on flat surfaces patterned with extracellular matrix proteins [41].

### Blastoids

Blastoids, on the other hand, are a more complex model that was developed to capture the development of the mammalian blastocyst. In the original protocol [35], embryonic and trophoblast stem cells were combined to give rise to implantation ready, blastocyst‐like structures. When mouse blastoids were transferred into pseudo pregnant mouse females, they were able to induce uterine decidualization and began implantation; however, to date, there are no reports of further embryonic development from blastoids *in utero* [45, 46, 47]. Since the original protocol, variations replacing the original stem cell ‘building blocks’ have been reported, for instance using ESCs with the potential to give rise to the other lineages [34, 48]. Despite these methodological improvements, blastoids, while displaying striking blastocyst morphology, appropriate gene expression, and the capacity to initiate implantation, still show low efficiency in acquiring postimplantation morphology *in vitro* and fail to develop following transfer into the uterus of pseudopregnant mouse females [45, 46, 48].

### ETiX‐embryoids

Similar to blastoids, ETiX‐embryoids (ETiXs) aim to reconstitute the cellular partnership existing within the mature blastocyst to capture the morphology and developmental potential of the mammalian embryo. For their formation, embryonic and trophoblast stem cells were combined with embryonic stem cells able to transdifferentiate into extraembryonic endoderm by transient expression of transcription factor GATA4 [28, 49]. Although ETiXs did not acquire blastocyst morphology, these stembryos developed over the course of 4 days to resemble mouse early postimplantation egg cylinders. ETiX culture could then be extended up to 8 days of development, allowing the structures to undertake gastrulation and early organogenesis, reaching a developmental stage roughly similar to mouse embryonic day (E) 8.75. To do so, ETiXs at 5 days of development were cultured on a roller culture incubator at controlled gas and pressure levels [28]. Similarities with mouse embryos extended beyond morphology and included comparable patterns of gene expression and formation of the appropriate cell types over time. Further protocol modifications have also been introduced to replace trophoblast stem cells with embryonic stem cells that adopt trophoblast fate by transient expression of transcription factor CDX2 [29, 50, 51]. At present, ETiX‐embryoids cannot develop beyond a stage equivalent to E8.5–E8.75, and the utilization of transgene‐free culture conditions to form the trophoblast and extraembryonic endoderm lineages has not improved developmental potential further [30, 52].

For all these models, human counterparts using human stem cells have also been established [32, 53, 54, 55], and primate embryo‐like structures have also been recently reported [42, 56]. Without a doubt, as permissible culture conditions for the isolation and propagation of embryonic stem cells from other mammalian species are discovered, similar models will also be developed for them.

## Advantages and disadvantages of stembryo models

Stembryos have the potential to become a transformational tool to understand mammalian development and they offer important advantages over traditional embryo work: First of all, their stem cell nature is amenable to rapid genetic manipulation [42, 43, 57]; secondly, the modular design of stembryos that combine multiple stem cell types enables simpler tissue‐specific modifications [36, 58, 59]. For instance, in ETiXs, a genetic modification to the placental lineage is introduced by modifying the TSCs, which will give rise to this lineage [49, 50, 51]. Third, stembryo protocols can be easily upscaled to generate scores of structures, thus eliminating the need for challenging embryo recoveries from pregnant females, while providing a large quantity of samples for experiments [38, 39, 58]. Finally, human stembryos, while not a replacement for human embryo research, are a more ethical alternative that can potentially be adopted also by countries in which human embryo research is outright prohibited [60].

Stembryos, however, are far from perfect and possess several limitations. First and foremost, there is a clear trade‐off between the overall efficiency of the system, its developmental complexity, and the different types of stem cells utilized. In other words, combining multiple types of stem cells yields more complex stembryo models that recapitulate more aspects of development, but it also results in an overall lower efficiency because not all the structures in a given experiment will develop correctly. On the other hand, a model with only one or two stem cell types will generally capture fewer aspects of development, but a much higher proportion of the structures in any given experiment will develop correctly [61, 62]. A second limitation is that very often stembryo development is assessed during an experiment by morphology, but this is not a reliable predictor of what structures will continue developing. In fact, structures that initially develop correctly may become aberrant; conversely, abnormal‐looking structures may correct themselves and resume normal development [63]. A third limitation is that well‐formed structures developing until the end of the culture protocol also display significant structure‐to‐structure variability or may have a bias in the cell types being generated [39, 63, 64].

Stembryo characterization has relied predominantly on morphological assessment and the analysis of lineage‐specific markers, often combined with transcriptomic profiling approaches such as single cell RNA sequencing (scRNAseq) [9, 59, 65]. These datasets are typically benchmarked against embryos at comparable stages of development to assess the presence, proportion, and transcriptional identity of expected cell types. More recently, approaches such as assay for transposase‐accessible chromatin using sequencing (ATAC‐seq) have also been utilized to evaluate regulatory chromatin accessibility and landscape similarities between stembryos and embryos [54]. Together, these approaches have revealed a remarkable degree of concordance at the level of morphology, cell type composition, and gene expression, while also highlighting model‐specific biases and variability.

Nonetheless, the difficulty in predicting successfully developing structures combined with the naturally occurring variability are significant confounding factors when, for instance, these models are utilized to characterize the function of novel genes, and this limits their applicability and usefulness. The causes leading to this experimental variability are not fully understood but could be underpinned by multiple factors. First and foremost, several protocols exist to derive and propagate embryonic and extraembryonic stem cells, which have profound effects on their potential to capture or recapitulate specific aspects of development [22, 24, 25, 66, 67, 68, 69, 70]. Second, stembryo culture conditions vary greatly among different protocols and these are also likely to affect stembryo long‐term development [69, 71, 72]. This lack of standardization, however, also means that ultimately there is no consensus on which conditions hold the greatest potential for long‐term stembryo development. Lastly, it is possible that existing protocols fail to capture additional fundamental aspects of embryonic development.

To help identify where important and potentially overlooked differences may lie, in this Perspective, we turn to the broad field of epigenetic regulation, with a particular focus on DNA methylation. DNA methylation is a crucial and tightly regulated feature of mammalian life, and its misregulation is frequently associated with lethality during embryonic development or disease during adulthood [73, 74, 75, 76]. Despite its well‐established importance in regulating the mammalian life cycle [77], the conservation and dynamics of DNA methylation during stembryo development have received comparatively little attention. Here, we suggest that the establishment of aberrant DNA methylation patterns during stembryo development may represent one contributing factor limiting their long‐term developmental potential and increasing their natural variability.

## DNA methylation and demethylation in early development

Epigenetics refers to the study of the mechanisms that regulate gene expression and cellular identity without altering the underlying DNA sequence, including, but not limited to, chemical modifications of DNA and histones, and regulatory RNA‐mediated processes. In this Perspective article, we specifically focus on DNA methylation as a well‐characterized and developmentally essential epigenetic layer.

DNA methylation is the most common form of DNA modification in mammals, and it consists in the addition of a methyl group to the fifth carbon of cytosine nucleotides. In the genome, it is present in both strands of CpG dyads and CpG islands (CpGIs), although the latter is nearly always unmethylated during development, and DNA methylation regulation has been widely shown to govern gene expression, chromatin accessibility, and DNA–protein interactions [78, 79, 80]. More specifically, DNA methylation is involved in a wide range of regulatory processes during mammalian development, including transcriptional regulation and gene silencing, X‐chromosome inactivation, repression of transposable and repetitive elements, control of viral integrations, and the establishment and maintenance of genomic imprinting [74, 81]. During mammalian development, several waves of demethylation and methylation occur, which are required to establish the correct, stage‐appropriate DNA methylation profile [82].

## DNA methylation regulators

The process of DNA methylation is controlled by DNA methyltransferase enzymes, which are classified depending on whether they interact with unmethylated or hemimethylated DNA. *De novo* DNA methyltransferases act on unmethylated DNA and are primarily active after demethylation steps, while maintenance DNA methyltransferases recognize hemi‐methylated DNA and are activated during DNA replication to preserve the methylation pattern in the newly synthesized strands [74, 78].

The most important maintenance DNA methyltransferase is DNMT1, which is characterized by a C‐terminal catalytic domain and an N‐terminal regulatory domain. Here, the replication foci targeting sequence (RFTS) inhibits DNMT1 activity by blocking the catalytic site until DNMT1 is recruited by the E3 ubiquitin‐protein ligase UHRF1, which specifically binds to hemi‐methylated DNA at the replication forks. UHRF1 recruits DNMT1 through its ubiquitin‐like (UBL) domain, causing the release of RFTS from the C‐terminal and enabling DNMT1 to bind histone H3 tails [75, 83].

*De novo* methylation is carried out by two major enzymes, DNMT3A and DNMT3B, which, similar to DNMT1, present a catalytic C‐terminal domain and a regulatory N‐terminal domain, though without sequence similarity to DNMT1. The regulatory region presents two important chromatin interacting domains: the proline–tryptophan–tryptophan–proline (PWWP), which binds to H3K36me3, a histone modification, and the ATRX‐DNMT3‐DNMT3L (ADD) domain that binds to K4 residues of H3 tails. The ADD domain facilitates methyl deposition until the K4 site accumulates methyl groups that repel the ADD sequence, leading to its binding on the catalytic site of the DNA methyltransferases [75, 84]. Alternative splicing of DNA methyltransferase genes produces regulatory protein isoforms that lack catalytic activity. Examples of this are DNMT3L, an accessory protein critical for *de novo* methylation in germ cells that works with DNMT3A, or DNMT3B4, which reduces the DNA‐binding ability of the DNMT3 enzymes by binding to them [85, 86].

DNA demethylation, on the other hand, can occur either passively or actively. Passive demethylation occurs when the methylation maintenance complex is inactive and results in the dilution of methylation through DNA replication [75, 87]. Active demethylation, instead, requires the activity of specific demethylases called ten‐eleven translocation (TET) enzymes. TET methylcytosine dioxygenases (TET1, TET2, and TET3) progressively oxidize 5mC to 5‐hydroxymethylcytosine (5hmC), 5‐formylcytosine (5fC), and 5‐carboxylcytosine (5caC), which then can be recognized and excised from DNA by thymine DNA glycosylase (TDG). Base excision repair events replace the abasic nucleotide with a normal cytosine. TET enzyme activity can also contribute to passive demethylation, as 5mC oxidized derivatives are not well recognized by the DNMT1 complex, facilitating their recognition as normal cytosines during DNA replication and treated as such [74, 88].

## DNA methylation and demethylation role in embryogenesis

Consistent with its central role in development, genetic disruption of DNA methylation and demethylation pathways in mice—most notably through loss of DNMTs or TET enzymes (see below)—results in severe developmental defects and early embryonic lethality.

During development, the first critical demethylation event happens in the germline, when maternal and paternal primordial germ cells undergo an initial global demethylation. As male primordial germ cells reach and colonize the gonadal ridge, they undergo methylation to establish parental and germline‐specific imprinting, whereas female primordial germ cells undergo this imprinting after birth. Following germline reprogramming, a second methylation reset takes place after fertilization. The newly formed zygote must acquire totipotency to build both embryonic and extraembryonic tissues and in this process most methylated regions are erased, with the exception of maternal and paternal imprinted loci, in which demethylation is prevented [74]. The maternal and paternal genomes are demethylated via different mechanisms. The paternal genome is actively demethylated through TET3‐mediated 5mC oxidation, whereas the maternal genome undergoes passive demethylation during DNA replication by active exclusion of DNMT1 from the maternal pronucleus [89]. Following implantation in the uterus, which marks the beginning of post‐implantation development, the embryonic germ layers are gradually specified and acquire the correct DNA methylation pattern, which becomes consolidated during somatic cell divisions, thus achieving stable inheritance [90, 91].

Methylation and demethylation enzymes must work together in a dynamic balance to maintain and regulate DNA methylation through the genome during embryo development, but DNMT1 specifically appears to have an essential role at these stages. In fact, DNMT1, together with UHRF1, is required for the maintenance of methylation at most imprinting control regions (ICRs) in mouse ESCs [92]. DNMT1 and UHRF1 are recruited by ZFP57, a KRAB zinc finger protein that, after recognizing the six‐nucleotide consensus motif TGCCGC present at almost all known ICRs, binds to the cofactor KAP1/TRIM 28, which then mediates the interaction with the methyltransferase complex [93, 94, 95, 96]. Liu et al. [92] observed that DNMT1 knockout (KO) in mouse ES cells resulted in the general complete loss of methylation at the examined ICRs, gene bodies, introns, and intergenic regions. Furthermore, KOs of DNMT1 and UHRF1 in mice are associated with early embryonic lethality after loss of DNA methylation, reflecting their essential primary functions during development [75, 77, 97].

DNMT3A/B, on the other hand, seem to have a more moderate role in maintaining methylation levels at imprinted regions compared to DNMT1 but are fundamental for establishing the new DNA methylation patterns post implantation [82, 98]. DNMT3B KO exhibits lethal phenotypes by midgestation, whereas DNMT3A KO causes mortality after birth. Moreover, DNMT3A germline‐specific KO in mice results in sterility in both females and males [75, 77, 97].

DNMT3L is dispensable for *de novo* methylation during embryogenesis, but quite important during DNA methylation establishment in germ cells, as an accessory protein for DNMT3A activity. Loss of function of DNMT3L in mouse development impairs meiosis in the male germline while female germ cells cannot establish maternal imprinting, leading to mid‐gestation lethality for the subsequent progeny [75]. DNMT3L‐deficient mouse ESCs revealed downregulation of DNMT3A and hypomethylation in many DNMT3A target regions [99]. Furthermore, loss of the functional ADD domain in DNMT3L causes a mild reduction of DNA methylation levels especially in mouse oocytes, and in maternally methylated ICRs, which is worsened when combined with DNMT3A loss of the ADD domain [100].

Interestingly, single KOs of TET1/2 in mouse models have not been associated with lethal phenotypes and are usually compatible with the development of viable embryos, which may suggest these TET family members may compensate for each other [101]. Consistently with this, complete loss of all three TET enzymes results in mid‐gestation embryonic lethality, likely reflecting a failure of global DNA methylation reprogramming [102]. Although not embryonic lethal, TET3 conditional KO has been reported to reduce fertility in the male germline, while complete KO is usually associated with perinatal death [75, 101].

The process of DNA methylation during embryogenesis is largely conserved between mice and humans, but differences are also present. For instance, human germ cells also undergo a genome‐wide DNA demethylation to a level similar to that of mice, but DNMT3L does not appear to be expressed in the human male germline [103]. Similarly, the female germline does not appear to express this gene, and it also exhibits a higher overall level of methylation in comparison to mouse oocytes [76].

DNA methylation also plays a fundamental role during reprogramming to induced pluripotency by stabilizing cell identity [104]. When somatic cells are reprogrammed to an induced pluripotent state, genome‐wide demethylation occurs in the final stages of this process, enabling the activation of pluripotency genes [105]. Consistently with this, KO of key DNA methylation regulators has been shown to impair the efficiency and fidelity of stem cell reprogramming [101, 106, 107].

## Placental methylation

An important feature of placental mammals, and thus one shared by mice and humans, is the acquisition of specific and distinct methylation signatures in the embryonic and extraembryonic tissues [90, 108, 109]. Epiblast cells, which give rise to the embryo proper, establish widespread genomic DNA methylation, and only active regulatory elements such as promoters and enhancers remain unmethylated. Trophoblast cells, and by extension the placenta, instead establish unique domains with partial levels of methylation over most of the genome, aptly named partially methylated domains. This so‐called placental methylome shows conservation across mammalian species and, after its establishment, is maintained for the duration of the pregnancy [110, 111]. Intriguingly, this unusual feature is also present in some types of cancers, implying a functional role in the context of gene regulation [109]. Altered methylation in the placenta, as well as altered levels of DNA methyltransferase enzymes [112], have been associated with pregnancy complications and miscarriages but also with long‐term health effects after birth [113].

## Outstanding gaps in DNA methylation during early embryogenesis

Despite the central role of DNA methylation and demethylation in development and cellular reprogramming, fundamental aspects of how these processes are regulated are still not fully understood. One major gap concerns the meaning of DNA methylation patterns, which can have different functional consequences depending on their surrounding context (for instance chromatin accessibility, histone modifications, transcription factor occupancy, developmental timing, and biological sex) [79]. Yet, DNA methylation is still mostly studied on its own, thus limiting our ability to predict when methylation changes are instructive, permissive, or merely reflective of underlying transcriptional states. Integration of DNA methylation analysis with other epigenetic modifications and chromatin states is needed to shed light on how they cooperate.

Another unresolved issue relates to causality in DNA methylation. While dynamic DNA methylation reprogramming is a hallmark of early embryogenesis, it remains unclear which methylation changes actively drive developmental transitions and which represent downstream stabilization of cell fate decisions. DNA methylation often follows transcription factor binding or histone mark deposition, raising the possibility that many reported methylation changes are secondary rather than developmental drivers [114, 115]. Temporally resolved, locus‐specific perturbation studies during defined developmental windows are required to directly test these relationships.

A further gap concerns the integration of DNA methylation dynamics with parental and environmental influences. Early embryos are exquisitely sensitive to changes in maternal physiology, which can influence DNA methylation in both imprinting control regions and regulatory elements [116, 117]. However, the mechanisms by which environmental cues intersect with these processes remain poorly understood, as do the long‐term developmental consequences of epigenetic perturbations. This lack of knowledge bears important implications for human health and reproduction, and especially in the context of assisted reproductive technologies (ART). While ART are generally considered safe and conducive to healthy pregnancies, there are also reports of long‐term adverse effects caused by the protocols and treatments to which gametes and embryos are subjected [118, 119]. As adoption of ARTs increases in the population, it will be important to elucidate these potential risks and devise novel approaches to minimize them.

## Is the long‐term development of stembryo models hindered by aberrant DNA methylation patterns?

As discussed earlier, mammalian embryogenesis is characterized by a highly orchestrated demethylation of the maternal and paternal genomes during pre‐implantation, with the exception of imprinting control regions, followed by *de novo*, lineage‐appropriate establishment of DNA methylation patterns during postimplantation development. By contrast, stembryo models are formed from the aggregation of pluripotent stem cells (PSCs), thereby bypassing this pre‐implantation zygotic reprogramming phase entirely. PSCs carry a pre‐existing DNA methylation landscape shaped by their derivation and maintenance *in vitro*, which may be affected by culture conditions [69, 71, 120], yet the fate of this epigenetic memory during aggregation and self‐organization remains largely unexplored. As a result, these models start from a DNA methylation ground state that is fundamentally distinct from that of the embryo, and the developmental consequences of this mismatch are still uncharacterised.

An additional unresolved issue concerns the faithful establishment of extraembryonic and placental‐specific DNA methylation patterns. In many stembryo models, including blastoids and ETiXs, some of the reported protocols generate extraembryonic lineages directly from pluripotent stem cells via transcriptional or chemical reprogramming rather than from *bona fide* trophoblast or primitive endoderm progenitors [29, 30, 34, 49]. Given the substantial differences in global methylation levels and patterns between pluripotent and trophoblast lineages, it remains unclear to what extent these PSC‐derived extraembryonic stem cells and tissues can acquire the correct DNA methylation signatures. To complicate matters further, recent findings showing that human trophoblast stem cells possess a methylome distinct from both trophectoderm and first‐trimester cytotrophoblast further underscore the complexity of this issue and indicate that also DNA methylation patterns of *bona fide* extraembryonic progenitors may be affected by derivation or long‐term culture conditions, thus warranting further investigation [121].

Finally, while stem cell reprogramming demonstrates that cell identity can be reversed through defined transcription factor expression and optimized culture conditions [122, 123], the epigenetic mechanisms affecting self‐organization in three‐dimensional embryo‐like aggregates are far less understood. In stembryo models, lineage specification emerges from collective signaling and spatial organization [124], thus raising unresolved questions about how intercellular signaling dynamically rewires DNA methylation and demethylation pathways in this context.

Together, these gaps highlight DNA methylation and demethylation not merely as descriptive features, but as potential mechanistic bottlenecks that may underlie the variability and limited developmental potential of current stembryo models.

## DNA methylation and stembryos: a missed opportunity?

In the following section, we review the relevant gastruloid, blastoid, and ETiX literature to seek evidence suggesting whether these models are able to establish stage‐appropriate DNA methylation patterns. The paucity of studies addressing this aspect in both gastruloids and blastoids, and the lack of studies in ETiXs, indicate an important knowledge gap that must be addressed. Because of the lack of DNA methylation data in ETiXs, we analyzed available single cell sequencing datasets, including our own, to examine the expression of the relevant DNA methyltransferase genes at different developmental stages (see *Data availability statement*). Altogether, while the initial evidence is far from definitive, and expression levels of DNA methyltransferase enzymes may not correlate with DNA methylation levels, we believe that the available data hints at the possibility that the establishment of DNA methylation patterns in these systems is incomplete, potentially constraining lineage stabilization, developmental robustness, and progression toward later stages. This issue may also be exacerbated by culture conditions, which are known to influence DNA methylation states [69, 71, 120], yet the lack of standardized protocols for stem cell and stembryo culture introduces additional variability and challenges reproducibility across laboratories.

### Gastruloids

In this recent work, Blotenburg *et al*., [71] sought to identify conditions yielding more homogenous gastruloids with respect to both elongation and cell contribution to different lineages. To achieve this, they examined different ESC culture conditions and characterized their gene expression, DNA methylation, and methylation of lysine 27 of histone 3 (H3K27Me3), prior to gastruloid formation. For a comprehensive overview on histone modification, readers are directed to these excellent reviews on the topic [114, 125], but in the context of this discussion, H3K27Me3 is thought to promote gene silencing and transcriptional repression [126]. The authors tested three mouse ESC lines from two different genetic backgrounds: E14 cells containing a triple germ layer reporter (TR), a wild‐type E14‐IB10 line (IB10), and wild‐type 129SV/Bl6 (B6) cells, which were treated with different media combinations to evaluate what culture conditions best support gastruloid formation. The authors utilized a medium containing fetal bovine serum and leukemia inhibitory factor (ESLIF) and a medium containing 2i. Consistently with other studies [106, 127, 128], they reported that different ESC culture conditions caused changes in pluripotency‐associated genes as well as DNA methylation and H3K27Me3 patterns. When these different lines were used for gastruloid formation, morphological screening of gastruloid elongation revealed interesting line‐specific effects: the TR line exhibited only modest elongation, and different pre‐culture conditions elicited no significant differences in gastruloid formation, unlike the B10 and B6 lines. Out of the different conditions tested, the B6 line displayed the highest elongation when it was cultured in ESLIF. Methylation analysis revealed high variability in H3K27Me3 and DNA methylation levels, especially at promoter regions, with transcriptionally active genes having relatively low H3K27Me3 levels. Notably, in the B6 line, the pre‐culture conditions that generated more reproducible gastruloids were associated with higher levels of DNA methylation regulators and H3K27Me3. Different pre‐culture conditions also influenced the cell types generated, with one condition favoring more ectodermal cells and two favoring mesodermal cells. Counterintuitively, however, they observed that gastruloids with a predominant mesodermal contribution had higher levels of repressive H3K27Me3 on mesodermal promoters. Unfortunately, for this particular experiment DNA methylation was not characterized, but it could have been informative. Furthermore, all these findings in ESCs and gastruloids were not compared to mouse embryos, which would have been helpful to establish a benchmark for further optimizations. Nevertheless, this study highlights an incomplete understanding of how DNA methylation states affect gastruloid developmental potential, and suggests a feasible experimental design to optimize ESC preculture and gastruloid culture conditions. It is also important to consider that the different genetic backgrounds of the diverse cell lines likely have an impact in the development of the models; hence, it may be beneficial to use cells of a well‐characterized genetic background when performing these studies.

### Blastoids

In the recent study by Xie *et al*., [129], in order to evaluate the quality of human blastoids generated by a novel method with 4CL‐naive ESCs, the authors assessed DNA methylation levels of both the starting ESC lines and their new blastoids and compared them to DNA methylation levels and patterns of human embryos and two of the most used blastoid models, 5iLA [130] and PXGL [32]. Their method was developed by supplementing the medium with a four‐chemical cocktail (chroman 1, emricasan, polyamines, and trans‐ISRIB (CEPT)), which mitigates stress mechanisms and enhances stem cell activity. The authors observed that 5iLA and PXGL human pluripotent stem cells (hPSCs) were characterized by low DNA methylation levels and loss of imprinted loci; moreover, the inner cell mass (ICM) and trophectoderm (TE) lineages from 5iLA and PXGL blastoids maintained low average DNA methylation levels in comparison to human blastocysts. On the other hand, 4CL‐naive hPSCs and blastoids' ICM and TE lineages derived from these cells displayed similar average DNA methylation levels compared to the corresponding cell types of the blastocysts and resulted in well‐formed structures. With respect to imprinting, allele‐specific methylation was not assessed; therefore, imprinting fidelity could not be conclusively established; however, these regions displayed DNA methylation patterns that were more similar to embryonic profiles. The authors also tested whether their blastoids could implant in an *in vitro* system and progress to postimplantation development until day 14. A subset of these blastoids was able to reach a stage similar to Carnegie stage 6 (CS6) of the human gastrula; however, the authors noted that their blastoids were missing some cell types present in human embryos and the fraction of structures undergoing morphogenetic events such as gastrulation remained low. It would have been very informative to analyze DNA methylation on the structures successfully developing until Day 14 and compare it to the structures that did not develop properly and also to human embryos, but these analyses were not performed. In the future, these comparisons could help elucidate why a fraction of structures fail to develop and how culture methods can be further improved. Nevertheless, this study is a good example of using human embryos to benchmark and evaluate different approaches to generate blastoids, and it is indeed showing that abnormal DNA methylation is present in two widely used approaches, suggesting that it may be a common limitation of existing protocols.

## Expression of DNA methyltransferase genes from published ETiX‐embryoid datasets

**Fig. 2:**
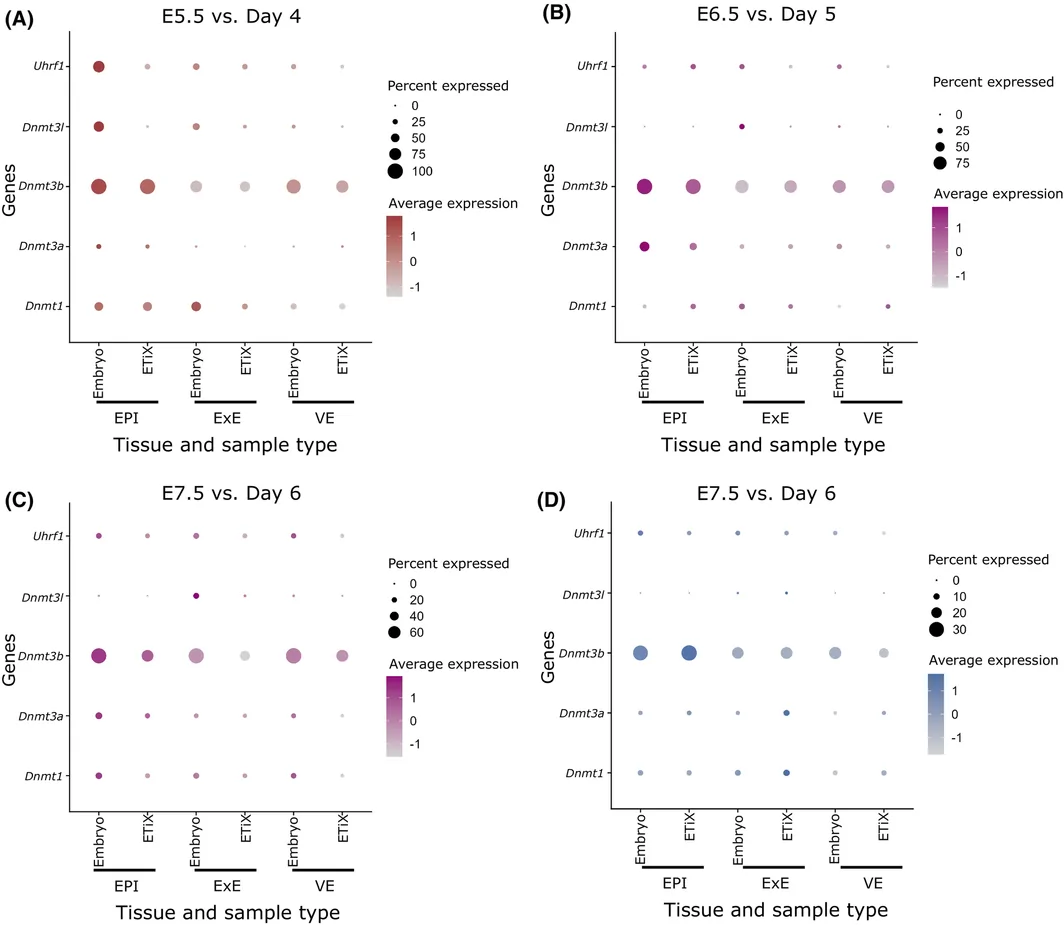
Analysis as dot plots of DNA methyltransferase enzyme gene expression in ETiX‐embryoids mouse embryos from several published single cell sequencing datasets: (A) in early post‐implantation (E5.5 vs. Day 4) [49]; (B) perigastrulation (E6.5 vs. Day 5) [28]; (C, D) late gastrulation (E7.5 vs. Day 6, InDrop and tiny‐SCI) [28]. Tissues examined comprise the epiblast, the extraembryonic ectoderm (ExE) and the visceral endoderm (VE), from embryos and ETiX.

**Fig. 3:**
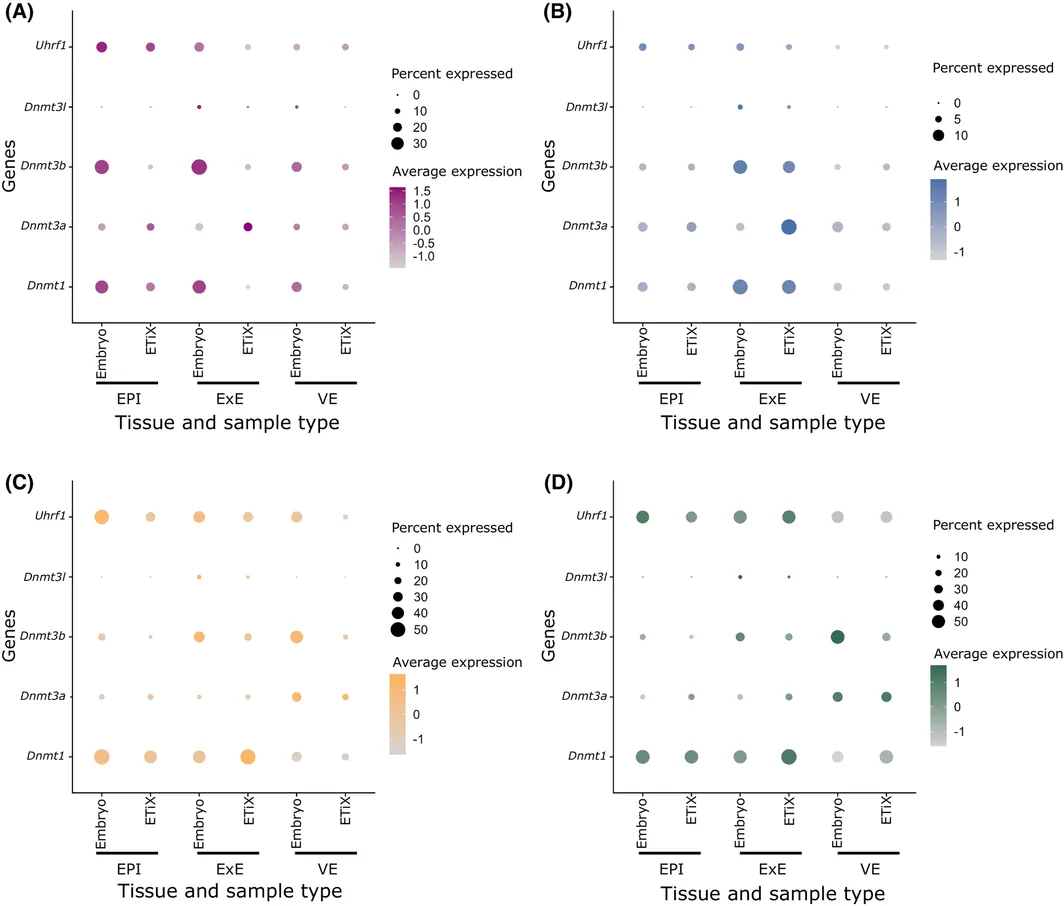
Analysis as dot plots of DNA methyltransferase enzyme gene expression in ETiX‐embryoids mouse embryos from several published single cell sequencing datasets at the early organogenesis stage around E8.5–8.75 vs. Day 8: (A) Amadei *et al*., InDrop [28]; (B) Amadei *et al*., tiny‐SCI [28]; (C) Tarazi *et al*., [29]; (D) Yilmaz *et al*., [30]. Tissues examined comprise the epiblast (Epi), the extraembryonic ectoderm (ExE) and the visceral endoderm (VE), from embryos and ETiX.

Since DNA methylation in ETiXs has not been examined, we analyzed single cell sequencing datasets to determine how the expression of DNA methyltransferase genes compares between mouse embryos and ETiXs (please see details in the *Data availability statement* section). Although expression of these genes is not a proxy for DNA methylation, we reasoned it may suggest whether more profound differences exist if we observed discrepancies between embryos and ETiXs. For this analysis, we examined available datasets published by us and others [28, 29, 30, 49]. These datasets were generated using different single cell sequencing methods: InDrop‐Seq in the case of Amadei *et al*., [28] (first dataset) and Amadei *et al*., [49], tiny‐single cell combinatorial indexing (tiny‐SCI) for Amadei *et al*., [28] (second dataset) and 10X Genomics Chromium for Tarazi et al. [29] and Yilmaz et al. [30]. These datasets comprise ETiXs from ESCs, TSCs and XEN cells generated by transient GATA4 overexpression [28, 49], ETiXs in which TSCs and XENs are generated by transient expression of CDX2 and GATA4 (sEmbryos, advanced postgastrulation embryo‐like models, in the original publication) [29], and ETiXs in which the extraembryonic lineages are generated by a chemical transdifferentiation approach (transgene‐free embryo models, TF‐SEM in the original publication) [30]. For our analysis (Figs 2 and 3), we examined the expression of DNMT1, DNMT3A, DNMT3B, DNMT3L and UHRF1 in the following tissues: the epiblast, which gives rise to the embryo proper, the extraembryonic ectoderm (ExE), which gives rise to the embryonic part of the placenta, and the visceral endoderm (VE), which gives rise to the yolk sac. At early postimplantation (Fig. 2A, E5.5 vs. Day 4), the expression of DNMT3A and DNMT3B appears fairly consistent between embryos and ETiXs. UHRF1 and DNMT3L instead appear to be more expressed in mouse embryos. DNMT1 is expressed consistently except in the embryo ExE, which shows higher expression. At the perigastrulation stage (Fig. 2B, E6.5 vs. Day 5), DNMT3A appears more expressed in the embryo epiblast, whereas DNMT1 appears to be more present in the ETiX epiblast and VE. Expression of DNMT3L is visible in the embryo ExE but it is not present in the ETiX ExE. Late gastrulation was analyzed in two different datasets (Fig. 2C,D, E7.5 vs. Day 6). In both datasets, we observed no strong differences in the expression of DNMT3A/B, DNMT1 and UHRF1, but in one dataset (Fig. 2C) DNMT3L and DNMT3B appeared to be more expressed in the embryo ExE and DNMT1 and DNMT3B may be more expressed in the embryo tissues. We then analyzed these genes at the early organogenesis stage for both embryos and ETiX (Fig. 3, E8.5/E8.75 vs. Day 8). While we did not observe completely concordant differences across all four datasets, DNMT3B appears to be consistently different between embryos and ETiX in most tissues (Fig. 3A,C,D), as does DNMT3A (Fig. 3A,B,D). DNMT3L expression seems to be confined to the ExE lineage and it is similar between embryos and ETiXs. DNMT1, with one exception, (Fig. 3A), seems to be expressed at similar levels in all datasets. This analysis, albeit a very preliminary one, suggests that differences in the expression of DNA methyltransferase genes may exist throughout ETiX development. The inconsistencies across datasets could be due to technical differences between single cell assays and ETiX protocols, and it would be beneficial to analyze the stembryos obtained from these different protocols with the same technical approaches to conclusively measure the expression levels of these enzymes and to assess DNA methylation. Measurements of DNA methylation levels can now be performed with increasingly sophisticated and sensitive sequencing approaches (Box 1). If these differences were confirmed, it would be interesting to speculate that reduced levels of DNMT1 and DNMT3A/B in ETiXs could impair both *de novo* and maintenance DNA methylation and could perhaps explain why ETiXs are unable to develop past E8.75. That is, without correct DNA methylation patterns, ETiX may fail to progress in their correct lineage specification and ultimately continue their development. It is also intriguing to observe that DNMT3L is mostly present in ExE/placental tissues of the embryo, but it is consistently downregulated in ETiXs. Although DNMT3L has been reported to be dispensable for a viable embryo, as long as it is maternally deposited in the oocytes, it is intriguing to speculate that its absence in ETiXs may explain why differentiated placental cell types are absent in this stembryo model [28]. Finally, it will be critical to determine whether these differences spontaneously arise during stembryo culture, or they are already present in the stem cell lines utilized for aggregation, or both.

Box 1 **DNA methylation profiling technologies.**

Although a wide range of technologies is available for DNA methylation profiling [131], at present their applications are not optimized for embryo models' analysis.

He *et al*., [132] categorized these techniques into two groups: bisulfite conversion‐based and bisulfite‐free methods. Whole‐genome bisulfite sequencing (WGBS) remains a gold standard technique for detecting DNA methylation in embryology, due to its genome‐wide, single‐base resolution, which also covers imprinting control regions and key regulatory elements [133]. However, it is costly, induces DNA degradation and requires a substantial amount of input material, which is one of the main limitations of early development models, where biological material is usually scarce. An important step in this direction has been taken by Derbala *et. al*., [134] who optimized WGBS for low‐input DNA samples.

Bisulfite‐free approaches including DNA immunoprecipitation followed by sequencing (MeDIP‐seq) [135] and methods based on methylation‐sensitive/−dependent restriction enzymes (MSREs/MDREs) [136, 137] partially address WGBS limitations as they are suitable for low‐input samples and preserve DNA integrity. Nevertheless, these methods are still less commonly used in embryology likely due to the presence of established protocols and data availability for WGBS [138, 139].

More advanced platforms such as Oxford Nanopore Technologies (ONT) Oxford, UK, and Pacific Biosciences (PacBio) Menlo park, CA, USA, are able to simultaneously detect DNA methylation, single‐nucleotide variants, and structural variations, but the complexity of data analysis and the higher costs that they require compared to WGBS still limit their use in embryology.

These technical considerations may be one factor explaining why many studies on embryo models have not yet considered epigenetics. The technical hurdles highlight the urgent need for approaches specifically adapted to the limitations and characteristics of embryo models and would benefit from optimization for a low amount of starting material and reduced application costs.

As mentioned at the beginning, while these differences have to be confirmed and they cannot be used as a proxy for DNA methylation, we believe that they suggest that morphological and transcriptional characterizations are but the tip of the iceberg to properly benchmark stembryo models, and much work will be needed in the future to fill these gaps and devise new and improved strategies for stembryo formation.

## Strategies to modulate DNA methylation patterns

Culture conditions may influence DNA methylation levels and in turn stembryo development, and this is consistent with what has been reported for embryonic stem cell culture. For instance, Choi *et al*., [120] reported that mouse ESCs cultured in 2iLIF over long term undergo irreversible changes to their DNA methylation profile. Specifically, they reported that a prolonged inhibition of the MEK pathway induces downregulation of UHRF1, DNMT1, DNMT3A, DNMT3B, and DNMT3L and genome hypomethylation, with progressive and irreversible demethylation of most of the imprinted control regions (ICRs). Substitution of MEK inhibitor PD0325901 with Src inhibitor CGP77675 rescued correct DNA methylation levels and preserved ICRs methylation, also over long‐term culture. This finding is very relevant because culture of mouse ESCs in 2iLIF is widely utilized, and therefore these alterations may also affect downstream stembryo protocols. Although they suggest that Src inhibition seems a viable alternative, they also report that culture with this inhibitor is not as effective in maintaining the proper naïve state of the embryonic stem cells, which instead acquire a more primed identity. Interestingly, MEK inhibition is also commonly utilized for naive hPSCs, which also show imprinting erosion under these conditions, suggesting that this may be a common issue [140]. Altered DNA methylation patterns in culture, however, are not common only to ESCs.

Lea *et al*., [121] reported that human trophoblast stem cell lines do not express the enzyme DNMT3L, which is instead robustly expressed in the human blastocysts and the trophoblast lineage from early placentas, and show differences in DNA methylation patterns. The authors partly rescued these differences by ectopic expression of DNMT3L but this strategy was not sufficient to restore methylation of imprinted loci; hence, improved culture conditions may be required to prevent their erosion.

Other strategies to alter DNA methylation levels could involve targeting DNA methyltransferase enzymes or their regulators. For example, RNA interference treatments could be used to decrease the expression of one or more proteins involved in DNA methylation regulation, inducing hypomethylation [141, 142], or SINEUP technology could be used to upregulate the translation of specific transcripts [143]. Alternatively, pharmacological approaches, such as specific epigenetic drugs, are becoming increasingly available. For instance, nucleoside analog inhibitors like azacytidine and decitabine have been used for therapeutic approaches for myelodysplastic syndrome (MDS) and myelomonocytic leukemia (CMML). At the molecular level, they are incorporated into DNA thanks to their similarity to cytosine, and inhibit the activity of DNMT enzymes, resulting in general hypomethylation. Instead, non‐nucleoside inhibitors, such as MG98 and hydralazine, inhibit enzymatic activity directly without being incorporated into the DNA [144].

In addition to drug treatments, targeted modulation of other biological processes can be potentially utilized to interfere with DNA methylation regulation. An example of this would be to interfere with sumoylation processes, since this has been shown to strongly impact DNA methylation and could be used to indirectly modify 5mc [145]. We surmise, then, that there will be other biological processes that could be exploited to indirectly change DNA methylation. For example, Quarto *et al*., [146] investigated the regulation between 5mC and m6A, which seem to be strongly associated.

However, as useful as these global approaches may be, they do not readily allow us to address the issue of causality, that is, are DNA methylation differences causative rather than merely correlative in development. To achieve this, directly testing whether altered DNA methylation at specific loci contributes to developmental failure in embryo models will require targeted perturbation approaches, such as locus‐specific epigenome editing at developmentally relevant regions. In this regard, technologies are now available specifically for this task. Policarpi *et al*., [147] used a catalytically inactive dCas9 fused with a trail array of GCN4 motifs, which act as binding sites for catalytically active single‐chained variable fragments (scFv)‐tagged DNA methylation regulators such as DNMT3A or DNMT3L which are then brought to specific regions by guide RNA scaffolds. Using this approach the authors increased DNA methylation up to 60% in *Col16a1* and *Hand1* promoters in mESCs. Likewise, Morita *et al*., [148] used a similar approach optimized to achieve DNA demethylation at specific target regions, by exploiting TET enzyme activity. The systematic utilization of these targeted approaches to tamper with and modulate DNA methylation on cell lines, stembryo and embryo models, will enable us to shed further light on how this epigenetic mark contributes to physiological development.

## Conclusion

Stembryos, new stem cell‐based embryo models, represent a promising breakthrough for studying mammalian embryonic development, offering many advantages such as ethical alternatives to embryos and amenability to genetic modifications and high‐throughput experiments. Unfortunately, these models possess important limitations, such as experimental variability and limited development, which are currently not fully understood. DNA methylation is an important and conserved mechanism of epigenetic regulation, involved in transcriptional regulation, transposon silencing, X chromosome inactivation, and genomic imprinting, and its role has been proven fundamental in regulating the different stages of embryonic development. However, the DNA methylation status of stembryo models is largely unknown. Expanding our knowledge of DNA methylation regulation during stembryo development is likely to provide important clues to understand the origin of this variability and limited developmental potential, and design novel strategies for further improvements. The potential reward for these efforts will be the development of stembryo models that are truly transformative.

## Author contributions

SC and GA wrote the paper together, with the help of CQ, who, together with AC and AS, also guided the computational analysis using Seurat.

## Data Availability

Datasets were obtained from the GEO DataSets repository for each study (Amadei *et al*., [49]; Amadei *et al*., [28]; Tarazi *et al*., [29]; Yilmaz *et al*., [30]) and processed in RStudio with Seurat for analysis and visualization as Dot Plots. Scripts used for data processing, along with details on the R version and packages employed, are available at the following GitHub repository: https://github.com/gianlucaamadei‐sketch/Epigenetic_Blind_Spots_scripts_code. The processed Seurat objects utilized for analyses are available from the corresponding author upon request.
